# Personal Protection Prior to Preoperative Assessment—Little more an anaesthesiologist can do to prevent SARS-CoV-2 transmission and COVID-19 infection

**DOI:** 10.1186/s42077-020-00066-x

**Published:** 2020-04-15

**Authors:** Vinodhadevi Vijayakumar

**Affiliations:** Consultant Anesthesiologist, Department of Anesthesiology and critical care, Meenakshi Hospital, Thanjavur, 613005 Tamil Nadu India

To the editor,

COVID-19 infection is transmitted by the pathogen SARS-CoV-2 and as of March 4, 2020, COVID-19 situation report 44 of World Health Organization (WHO), 93,090 confirmed cases are reported, and 76 countries outside china have been affected (WHO 2020). It has a mortality rate of 4.3% in the initial case series from China (Wang et al. 2020). Anesthesiologists playing the role as an emergency physician, perioperative physician and intensivists, are at high risk of exposure and acquiring the disease. The recommendations from the renowned anaesthesiology societies are on personal protective equipment (PPE) and precautions to be taken during the intubation and transportation of such patients (Coronavirus (2019-nCoV) n.d.; Perioperative Considerations for the 2019 Novel coronavirus (COVID-19) n.d.; Peng et al. 2020). In high-income countries, the patients encountered by anaesthesiologists would have already been suspected or confirmed as suffering from COVID-19 and might have the facility for such PPEs. There is a severe shortage of supply of PPEs endangering health workers worldwide as reported by WHO on March 3, 2020 (Shortage of personal protective equipment endangering health workers worldwide n.d.). WHO reports the shipping of nearly half a million sets of personal protective equipment to 47 countries, but supplies seem to be rapidly depleting (Shortage of personal protective equipment endangering health workers worldwide n.d.).

Based on WHO modelling, an estimated 89 million medical masks are required for the COVID-19 response each month. For examination gloves, that figure goes up to 76 million, while international demand for goggles stands at 1.6 million per month (Shortage of personal protective equipment endangering health workers worldwide n.d.). Considering the exponential rise in demand and limited supply of the PPEs, the WHO has recommended the rationale use of PPEs in COVID-19 (Rational use of personal protective equipment for coronavirus disease 2019 (COVID-19) n.d.).

In the low- and middle-income countries (LMIC), depending on the health care system, anaesthesiologists may be the first person to suspect COVID-19 in a patient. Apart from the patient, the patients’ relatives/bystanders may be a source of infection. Individual anaesthesiologists should be determined for personal protection depending on the resource availability, may it be a simple surgical mask and handwashing with soap. Specifically, during preoperative assessment to enquire upon is recent travel (< 2 weeks) to the countries affected by COVID-19 (WHO 2020), by the patient himself or his family members, neighbours or colleagues. Exposure to such persons, even if the patient is asymptomatic currently, has to be elicited before proceeding with the clinical examination.

Apart from the operation theatre and intensive care unit, while administering anaesthesia in remote locations like endoscopy rooms, cardiac catheterisation labs, magnetic resonance imaging suites, recommended precaution has to be taken (Rational use of personal protective equipment for coronavirus disease 2019 (COVID-19) n.d.)

Resources for personal protection may vary depending on the health care system and the country, but the health care workers have to use the facilities locally available. It is the nonadherence to personal protection which has caused disease in health care professionals inspite of the availability of PPEs in the past (Peng et al. 2020). Anaesthesiologists should create awareness according to WHO guidelines on PPEs (Rational use of personal protective equipment for coronavirus disease 2019 (COVID-19) n.d.) and, as a team leader, ensure the adherence to personal protective measures by all health care workers in the team. I strongly emphasise “personal protection prior to preoperative assessment”, even before the patients’ arrival to operation theatre for anaesthesia, to decrease the transmission of SARS-CoV-2 and COVID-19.

## Data Availability

Not applicable

## Abbreviations

COVID-19: Coronavirus disease – 2019
LMIC: Low- and middle-income countries
PPE: Personal protective equipment
SARS-CoV-2: Severe adult respiratory syndrome - coronavirus - 2
WHO: World Health Organization
